# Effect of *Nigella sativa* and its bioactive compound on type 2 epithelial to mesenchymal transition: a systematic review

**DOI:** 10.1186/s12906-019-2706-2

**Published:** 2019-10-30

**Authors:** Abid Nordin, Haziq Kamal, Muhammad Dain Yazid, Aminuddin Saim, Ruszymah Idrus

**Affiliations:** 1 Department of Physiology, Faculty of Medicine, Universiti Kebangsaan Malaysia, 56000 Cheras, Kuala Lumpur Malaysia; 2 Nordin Kamil Consulting, 30-2, Jalan Dwitasik, Dataran Dwitasik, 56000 Cheras, Kuala Lumpur Malaysia; 3 Tissue Engineering Centre, Faculty of Medicine, Universiti Kebangsaan Malaysia, 56000 Cheras, Kuala Lumpur Malaysia; 4 Ear, Nose & Throat Consultant Clinic, Ampang Puteri Specialist Hospital, 68000 Ampang, Selangor Malaysia

**Keywords:** *Nigella sativa*, Thymoquinone, Epithelial, Mesenchymal, Wound healing, Fibrosis

## Abstract

**Background:**

*Nigella sativa* or commonly known as black seed or black cumin is one of the most ubiquitous complementary medicine. Epithelial to mesenchymal transition (EMT) of type 2 is defined by the balance between wound healing and tissue fibrosis, which is dependent to the state of inflammation. This systematic review is conducted to provide an overview regarding the reported effect of *Nigella sativa* and its bioactive compound on the type 2 EMT.

**Methods:**

A search was done in EBSCOHOST, OVID and SCOPUS database to obtain potentially relevant articles that were published between 1823 and August 2019. This review includes studies that focus on the effect of *Nigella sativa* and its bioactive compound on the events related to type 2 EMT.

**Results:**

A total of 1393 research articles were found to be potentially related to the effect of *Nigella sativa* and its bioactive compound, thymoquinone on Type 2 EMT. After screening was done, 22 research articles met inclusion criteria and were included in this review. Majority of the studies, reported better wound healing rate or significant prevention of tissue inflammation and organ fibrosis following *Nigella sativa* or thymoquinone treatments. In terms of wound healing, studies included reported progression of EMT related pathological changes after treatment with *Nigella sativa* or thymoquinone. Alternatively, in terms of fibrosis and inflammation, studies included reported reversal of pathological changes related to EMT after treatment with *Nigella sativa* or thymoquinone.

**Conclusion:**

Through this review, *Nigella sativa* and thymoquinone have been associated with events in Type 2 EMT. They have been shown to promote wound healing, attenuate tissue inflammation, and prevent organ fibrosis via regulation of the EMT process.

## Background

### Epithelial to mesenchymal transition

The term epithelial-to-mesenchymal transition (EMT) was first coined by Elizabeth Hay to describe the phenotypical transition of epithelial cells into mesenchymal cells during the primitive streak formation in chick embryo [1]. This transition is a ubiquitous physiological process that plays a major role in healing process and disease progression [2].

EMT resulted in the release of the immotile epithelial cell that was bounded to their basal membrane, into a non-polarized cell that is freely mobile [3]. The transition is further reinforced by increased production of extracellular matrix (ECM) components [4]. At the molecular level, the EMT process is regulated by several molecular events such as transcription factors activation, cytoskeletal reorganization, and specific cell-surface proteins or microRNAs regulation [5].

### Classification of EMT

EMT classification has been made according to their functional consequences; namely type 1, type 2, and type 3 [4]. Type 1 EMT is assigned to the embryological development, implantation and organ development. It is involved primarily during gastrulation and neurulation, whereby the cell transition between the two different state of epithelial and mesenchymal as needed [6]. These cells might be re-induced further as secondary epithelial cells that were involved in organogenesis and development [7]. Type 2 EMT is involved primarily in tissue regeneration [8] and fibrosis [9]. This happens when inflammation initiate the production of ECM by the fibroblast, to help rebuild the damaged tissue site [10]. However, persistent inflammation can lead to tissue or organ fibrosis following incessant wound healing process [11]. Hence, the balance between wound healing and fibrosis via the inflammation process is what defines the type 2 EMT. On the other hand, type 3 EMT, has been specifically assigned for neoplastic cells in regards to their invasive capability seen in their metastasis to secondary site from their primary oncogenic origin [12].

### Markers for EMT

In 2009, a review was made by Michael Zeisberg and Eric G. Neilson regarding biomarkers of EMT [13]. The loss of the epithelial marker, E-cadherin, and the increase expressions of mesenchymal marker, N-cadherin, together known as the cadherin switch, has been nominated as the hallmark of EMT process [14]. The transition into mesenchymal phenotype was also indicated by the elevation of other mesenchymal markers such as fibroblast specific protein-1 (FSP1), discoidin domain receptor tyrosine kinase 2 (DDR2) or fibronectin [15]. Activation of EMT also triggers the production of matrix metalloproteinase [16]. This results in breaking down of the cell-to-cell adhesion that lead to the increased migratory capability of the resulting mesenchymal cell [13].

Following the advancement of molecular biology, a review on the molecular markers of EMT was made by Samy Lamouille, Jian Xu and Rik Derynck in 2014 [5]. They described regulators of EMT at the transcriptional, translational and post-translational level. In terms of transcriptional factors, some important characters are SNAIL, TWIST and ZEB family of transcription factors [5]. Two variants of SNAIL, SNAI1 (Snail) and SNAI2 (Slug) reduce the expression of E-cadherin by repressing its promoter [17]. In cancer cell, activity of TWIST1 has been shown to have dual transcriptional activities, namely the repression of E-cadherin and activation of N-cadherin [18]. Finally, like SNAIL and TWIST, ZEB transcription factors also bind to E-cadherin promoter and function as transcriptional repressors of E-cadherin protein [19].

### *Nigella sativa* and its bioactive compound in type 2 EMT

*Nigella sativa* or commonly known as black seed or black cumin is one of the most ubiquitous complementary medicine. It is widely grown in the Middle East, Africa and Asia [20]. In Islam, it was mentioned by the prophet Muhammad (PBUH) for its healing properties [21] and currently widely used in the Islamic practice of prophetic medicine [22]. Pre-clinical and clinical studies have shown *Nigella sativa* to have multiple health benefits such as anti-inflammatory [23], anti-oxidant [24], anti-bacterial [25], anti-angiogenic [26], and organ-protective [27].

On the other hand, a study on *Nigella sativa*’s chemical composition by supercritical carbon dioxide revealed few bioactive compounds; namely thymoquinone, dithymoquinone, and dihydrothymoquinone. Among these compounds, thymoquinone is the major active compound in *Nigella sativa* [28]. Thymoquinone by itself exhibit strong anti-inflammatory [23], anti-bacterial [25], anti-diabetic [26] and anti-oxidant properties [29].

Type 2 EMT plays a role in wound healing and tissue fibrosis, which is dependent to the state of inflammation. Inflammation is a complex mechanism that are influenced by bacterial activity [25], inflammatory response [30] and oxidative stress [29]. Balance between the attenuation and sustenance of these factors determine the fate of the tissue. On one hand, wound healing progresses with attenuation of inflammation. Alternatively, tissue fibrosis progresses with the sustenance of inflammation [31]. In this review, a systematic search of the electronic databases was conducted to identify studies done on different events of EMT type 2, including wound healing, tissue inflammation and organ fibrosis treated with *Nigella sativa* or thymoquinone.

## Methods

### Literature search/search strategy

A systematic review of the literature was conducted to identify relevant reports on the effects of *Nigella sativa* and its bioactive compound on the EMT, particularly in type 2 EMT. A comprehensive search of biomedical science journals in Ebscohost (published between 1823 to August 2019), Ovid (published between 1946 to August 2019) and Scopus (published between 1973 to August 2019) databases was done. The search strategy was adapted from the previous publication looking at the effect of honey in type 2 EMT [32] involving a combination of the following two sets of keywords; (1) thymoquinone OR *Nigella sativa* OR dithymoquinone OR thymohydroquinone AND (2) epitheli* OR mesenchym* OR transition OR vimentin OR cadherin OR tgf*.

### Inclusion criteria

Only original research articles (in vitro, in vivo or clinical study) that discuss changes in epithelial to mesenchymal transition parameters (down regulation of epithelial markers and up regulation of mesenchymal marker) of type 2 (wound healing, inflammation or tissue fibrosis) influenced by *Nigella sativa* or thymoquinone, written in English language and have abstract available were included in this review. Studies included must measure the changes of at least one of the epithelial or mesenchymal markers such as 1) cadherin switch; 2) elevation of FSP1, DDR2, or fibronectin; or 3) increased activity or expression of the metalloproteinase.

### Exclusion criteria

All secondary literatures and any original articles that were not written in English, do not have abstract available and do not fulfil the inclusion criteria mentioned were excluded. Studies focusing on other types of EMT such as type 1 (embryonic development) and type 3 (cancer metastasis) were excluded from the review.

### Data extraction and management

Articles underwent screening process prior to their inclusion in this review. Titles and abstracts were screened first to ensure inclusion and exclusion criteria were adhered. Then, the full text of what remaining were read thoroughly by five independent reviewers to exclude any article that did not meet inclusion criteria. All reviewers must agree on the inclusion of selected articles for the review before the data extraction phase begins. Any differences in opinions were resolved through discussion between the reviewers.

Data extraction was performed independently with the use of a data extraction form. The following data were recorded from the studies: (1) type of experimental model used; (2) treatment groups; (3) summary of the outcomes measured in the study; (4) summary of the study results; and (5) conclusion of the study.

## Results

### Search results

**Fig. 1:**
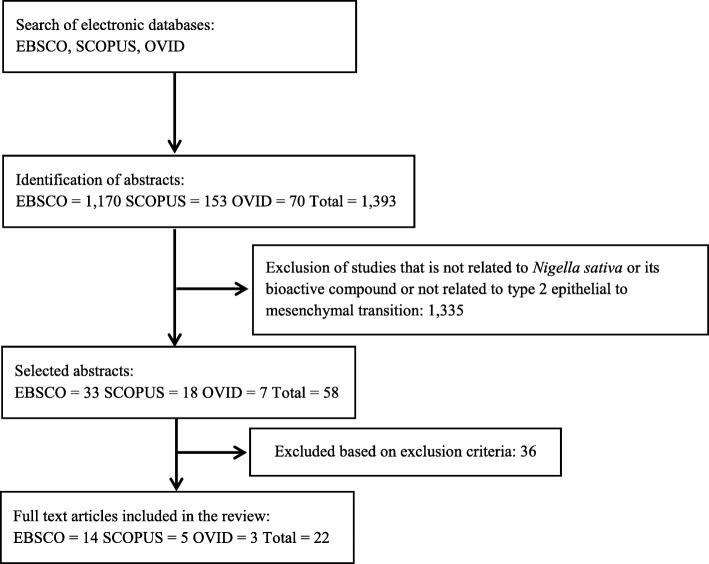
Flowchart of the selection process

The literature search identified 1393 potentially relevant articles. Four reviewers independently assessed all articles for inclusion and exclusion based on title and abstract. One thousand three hundred thirty-five of these articles were excluded because they were not related to *Nigella sativa* or its bioactive compound or not related to EMT in wound healing, inflammation, or tissue fibrosis. From the remaining 58 articles, 36 articles were rejected based on exclusion criteria after reading their full text. A total of 22 articles were retrieved for further assessment and data extraction to be included in this review. A flow chart of the selection process, including reasons for exclusion was shown in Fig. 1.

### Study characteristics

There are 22 articles that were included in the review. All articles were published between the year 2010 and 2018. The studies were categorized according to the type of pathophysiology that they model which were wound healing, inflammation and organ fibrosis.

*Nigella sativa* was used as oil ointment in five studies [23, 24, 27, 33, 34], and as cream in one study [35]. Thymoquinone was used in 14 studies [25, 26, 29, 36–46]. *Nigella sativa* oil and thymoquinone was compared in one study [47]. Since honey made by bee contains the bioactive compound of its nectar origin, a study that investigated the effect of *Nigella sativa* honey and its propolis was also included in the review [48].

Wound healing of two types of tissue were modelled among the studies included, cutaneous tissue and corneal tissue. In terms of cutaneous tissue, three types of wound model were used, namely incisional wound, burn wound, and diabetic wound. The two studies that investigated the incisional wound physiology uses mouse [25] and rat [35]. For burn wound, three studies using rat were included [29, 34, 48]. Only one study investigated the cutaneous diabetic wound [26]. In terms of corneal wound healing, one study using rat was included [27]. Studies reporting effect of *Nigella sativa* or thymoquinone on wound healing were listed in Table 1.

**Table 1:** Effect of *Nigella sativa* and thymoquinone in wound healing

References	Experimental model	Treatment	Outcome measures	Results	Conclusion
Cutaneous Incisional Wound					
Gomaa et al. 2017 [25]	Incisional wound in Balb/C mice.	3% (w/w) thymoquinone (TQ) loaded into nanofiber.	1. Wound area measurement. 2. Histopathology assessment.	Treatment with TQ improve wound closure and tissue healing.	TQ-loaded nanofiber shows potential to be used for wound dressing.
Han et al. 2017 [35]	Full thickness wound in Wistar rats.	50% topical *Nigella sativa* (NS) cream.	1. Wound contraction. 2. Biochemical analysis. 3. Histological evaluation.	Treatment with NS cream increases wound contraction rate and antioxidant activity but have no effect in tissue granulation.	NS heals via its antioxidant effect in full thickness wound.
Cutaneous Burn Wound					
Sulaiman et al. 2014 [48]	Burn wound model in albino rats	Topical micro-emulsion of 5% NS honey and 5% of its propolis.	1. Wound contraction.	Treatment of NS honey and its propolis increases wound contraction rate.	Microemulsion of NS honey and propolis contribute to faster burn wound healing.
Selcuk et al. 2013 [29]	Burn wound model in Sprague-Dawley rats	2 mg/kg/day oral TQ or 0.5% topical TQ.	1. Histological assessment. 2. Total antioxidant state. 3. Total oxidative stress. 4. Bacterial assessment	Topical treatment of TQ was superior compared to oral TQ in improving wound histology, enhancing antioxidant activity, and reducing bacterial growth.	Topical TQ is superior to oral TQ in improving wound healing.
Yaman et al. 2010 [34]	Burn wound model in male, Wistar-albino rats.	50% topical NS oil (NSO).	1. Gross morphology of the wound. 2. Histological evaluation.	Treatment with NSO reduces inflammation and demonstrated better tissue granulation in wound.	NSO has been shown to promote faster burn wound healing.
Cutaneous Diabetic Wound					
Yusmin & Ahmad 2017 [26]	Chronic delayed wound in alloxan-induced diabetic rats.	10% topical TQ.	1. Wound contraction. 2. Histological evaluation.	Treatment with TQ increased healing and reduced inflammatory cells and fibroblast at day 3. However, wound improvement declines on day 7 and day 14.	TQ heals faster in inflammatory phase but slower during the proliferative phase due to its antiangiogenic properties
Corneal Wound					
Salem et al. 2016[27]	Formaldehyde-induced corneal toxicity in albino rats.	40 mg/kg/day oral NSO	1. Histological evaluation.	Treatment with NSO reverses formaldehyde-induced pathological changes.	NSO shown to resolved corneal injury induced by formaldehyde toxicity

In terms of tissue fibrosis, 5 studies that model the tissue fibrosis of different organs were included. For cardiac fibrosis, one study using doxorubicin-induced rats was included [38]. For lung fibrosis, 2 studies with bleomycin-induced rats [23] and paraquat-induced rats [42] were included. For liver fibrosis, one study using carbon tetrachloride-induced rats was included [40]. For renal fibrosis, one study using nitrite-induced rats was included [24]. Studies reporting effect of *Nigella sativa* or thymoquinone on tissue fibrosis were listed in Table 2.

**Table 2:** Effect of *Nigella sativa* and thymoquinone on organ fibrosis

References	Experimental model	Treatment	Outcome measures	Results	Conclusion
Myocardial Fibrosis					
Pei et al. 2018 [38]	Doxorubicin (Dox)-induced heart failure in Sprague-Dawley rats.	50 mg/kg/day oral TQ.	1. Left ventricular functions. 2. Atherosclerotic lesion. 3. Fibrosis markers. 4. Apoptosis markers.	Treatment of TQ reverses Dox-induced pathological changes in the heart via inhibition of fibrosis and apoptosis.	TQ mitigates Dox-induced cardiac damage and fibrosis.
Pulmonary Fibrosis					
Abidi et al. 2017 [23]	Bleomycin-induced pulmonary fibrosis in Wistar rats.	1 mg/kg/day oral NSO.	1. Physical measurements. 2. Histological evaluation. 3. Liver metabolites. 4. Urine metabolites. 5. Expression of TGF-β1.	Treatment with NSO reverse bleomycin-induced pathological changes via induction of TGF-β1.	NSO have shown to resolve BLM-induced PF due to its anti-inflammatory and anti-fibrotic properties
Pourgholamhossein et al. 2016[42]	Paraquat-induced lung fibrosis in NMRI mice.	20 mg/kg/day and 40 mg/kg/day oral TQ.	1. Histological evaluation. 2. Oxidative stress analysis. 3. Hydroxyproline content. 4. Gene expression.	Treatment with TQ reverses paraquat-induced lung fibrosis inhibition of fibrosis and antioxidant activity.	TQ is able to reduce pulmonary fibrosis via its anti-fibrotic property.
Liver Fibrosis					
Abdelghany et al. 2016 [40]	Carbon tetrachloride (CCl_4_)-induced renal fibrosis in Wistar rats.	15 mg/ml oral TQ with or without 1000 IU/ml of Vitamin D3.	1. Liver function parameters. 2. Renal function parameters. 3. Histological assessment. 4. Cytokines level.	Treatment of TQ reverses CCl4-induced renal fibrosis via inhibition of inflammation.	TQ shows anti-fibrotic properties in carbon tetrachloride-induced renal fibrosis.
Renal Fibrosis					
Al-Gayyar et al. 2016 [24]	Sodium nitrite (NaNO_2_)-induced renal fibrosis in Sprague-Dawley rats.	2.5 ml/kg oral NSO.	1. Renal function parameters. 2. Fibrotic markers. 3. Cytokine levels. 4. Protein kinase activity. 5. Apoptosis markers.	Treatment with NSO reverses sodium nitrite-induced renal fibrosis via antioxidative, anti-inflammatory, and anti-apoptotic properties.	NSO have been shown to resolve NaNO_2_-induced nephrotoxicity.

Progression of wound healing and tissue fibrosis rely on the inflammation state of the tissue [31]. As a result, type 2 EMT researchers focus on the inflammatory properties of the diseased tissue. In terms of inflammation, 11 studies that model inflammation in different organs were included. In terms of the nose inflammation, rhinosinusitis, one study using rats induced with intranasal platelet activation factor was included [46]. Ear inflammation, otitis, is modelled in one study using bacterial infection-induced model [36]. In terms of inflammation following lung injury, 3 studies were included using cardiac ischemia-induced rats [39], cadmium chloride-induced rats [33], and ovalbumin-induced rats [43]. In terms of inflammation in the kidney, 2 studies using diabetic-induced nephropathy model in rats [45, 47] and one study using reperfusion therapy-induced nephropathy in rats [41] were included. One study focussing on liver inflammation using ethanol-induced liver injury model in mice [44] was included. Injury to testicle was modelled in rats in one study [37]. Studies reporting effect of *Nigella sativa* or thymoquinone on inflammation were listed in Table 3.

**Table 3:** Effect of *Nigella sativa* and thymoquinone on inflammation

References	Experimental model	Treatment	Outcome measures	Results	Conclusion
Rhinosinusitis					
Cingi et al. 2011 [46]	Intranasal platelet activating factor-induced rhinosinusitis model in Sprague-Dawley rats.	80 mg/kg oral TQ.	1. Degree of vascular congestion 2. Intensity of inflammatory cell infiltration 3. Degree of epithelial injury	Treatment with TQ reverses the intranasal platelet activating facto-induced histological changes.	TQ have been shown to be beneficial for the resolution of rhinosinusitis.
Otitis					
Demirel et al. 2018 [36]	Bacterial infection-induced otitis model in Wistar rats.	0.1 and 0.4% topical TQ.	1. Histopathological assessment. 2. Bacterial assessment.	Treatment of TQ reverses infection-induced histopathological changes and reduces bacterial growth in the ear canal.	TQ shows bacteria eradication and anti-inflammatory properties
Lung Inflammation					
Sezen et al. 2018 [39]	Cardiac ischemia-induced lung injury in Wistar rats	25 mg/kg TQ.	1. Apoptotic markers in the lung. 2. Histological assessment.	Treatment of TQ reverses cardiac ischemia-induced histopathology changes via suppression of apoptosis.	TQ protects against lung injury via inhibition of apoptosis.
El-Ebiary et al. 2016 [33]	Cadmium chloride (CdCl_2_)-induced lung damage in Wistar rats	1 ml/kg oral NSO.	1. Histopathological assessment. 2. Scanning electron microscopy.	Treatment of NSO reverses histopathological changes induced by CdCl_2_ with normal pneumocytes morphology and intra-alveolar septum thickness.	Treatment with NSO ameliorated pathological changes in CdCl_2_ poisoned rats.
Su et al. 2016 [43]	Ovalbumin-induced asthma in Balb/C mice.	3 mg/kg oral TQ.	1. Cytokines level. 2. Fibrotic markers. 3. Histopathological assessment. 4. Angiogenic factors. 5. HUVEC tube formation. 6. Protein kinase activity.	Treatment of TQ reverses histopathology changes of asthma induced by ovalbumin via suppression of inflammation and angiogenesis.	TQ have anti-inflammatory and anti-angiogenesis properties in ovalbumin-induced asthmatic mice.
Nephropathy					
Al-Trad et al. 2016 [47]	Streptozotocin (STZ)-induced nephropathy in diabetic rats.	50 mg/kg oral TQ and 2 ml/kg oral NSO.	1. Renal pathology parameters. 2. Expression of Podocin. 3. Fibrotic markers. 4. Angiogenic marker.	Both TQ and NSO treatment demonstrated comparable reversal of diabetic-induced renal pathology via expression of podocin and inhibition of fibrosis and angiogenesis.	TQ & NSO improves pathological changes in diabetic-induced nephropathy.
Omran 2013 [45]	Nephropathy in STZ-induced diabetic rats.	50 mg/kg oral TQ.	1. Renal pathology parameters. 2. Histopathological assessment. 3. Epithelial markers. 4. Mesenchymal markers.	Treatment of TQ reverses the diabetic-induced renal histopathological changes via inhibition of the epithelial to mesenchymal transition.	TQ improves renal functions via inhibition of epithelial to mesenchymal transition in diabetic nephropathy.
Hammad & Lubbad 2016 [41]	Reperfusion therapy-induced nephropathy in male Wistar rats.	10 mg/kg oral TQ.	1. Renal pathology parameters. 2. Cytokine levels.	Treatment of TQ resulted reversal of reperfusion therapy-induced histopathological changes via the inhibition of inflammation.	TQ improves renal functions via inhibition of inflammation following reperfusion therapy-induced nephropathy.
Liver Inflammation					
Yang et al. 2016 [44]	Ethanol (EtOH)-induced liver injury in C57/BL6 mice.	20 mg/kg or 40 mg/kg oral TQ.	1. Liver pathology parameters. 2. Histopathological assessment. 3. Expression level of SIRT1, LKB1 and AMPK.	Treatment of TQ reverses the EtOH-induced liver pathological changes via upregulation of SIRT1, LKB1, and AMPK.	TQ regulates LKB1 and AMPK signalling that is associated with inflammation in ethanol-induced liver injury.
Testicular Damage					
Mabrouk 2018 [37]	Lead (Pb)-induced testicular damage in Wistar rats.	5 mg/kg/day oral TQ.	1. Testicular pathology parameters. 2. Histopathological assessment.	Treatment of TQ reverses the Pb-induced testicular pathological.	TQ have protective effect against Pb-induced testicular damage.

### Potential of *Nigella sativa* and thymoquinone in wound healing

#### Cutaneous normal wound healing

The literature search returns some evidence on the positive effect of *Nigella sativa* and its constituent on cutaneous wound healing. The similarities of molecular events surrounding epithelial to mesenchymal transition and wound healing has been well described in the context of cutaneous wound [8]. Phenotypic changes following EMT in cutaneous tissue enable the progression of reepithelialisation of the epidermal layer in normal wound healing [49].

In incisional wound, the positive effect of topical application of *Nigella sativa* cream (NSC) on full thickness wound healing in rats was observed. The NSC-treated groups demonstrated the smallest wound size by the end of experiment (99.69% wound area reduction) compared to the control groups (81.35% wound area reduction). Histologically, NSC also reduces inflammatory cell infiltration, and lessen cell oedema. Highest antioxidant levels were also observed in NSC-treated rat’s tissues suggesting the correlation between *Nigella sativa* antioxidant effect and its wound healing benefits [35].

Delivery of thymoquinone, via the thymoquinone-loaded composite nanofiber, revealed a positive outcome to incisional wound healing in mice model. Macroscopically, application of thymoquinone-loaded dressing on mice wound produced the best wound closure. Histologically, the treatment group shows higher granulation tissue thickness in a shorter time compared to the control group. It also shows highest epithelial migration after 14 days. Thymoquinone delivery also resulted in the highest collagen deposition in the healing tissue via Masson’s Trichrome staining. This study concludes that thymoquinone promotes better wound healing via its anti-inflammatory capabilities [25].

Taken together, both *Nigella sativa* and its bioactive compound, thymoquinone, proves to be efficacious in enhancing the progression of incisional wound healing.

#### Cutaneous burn wound healing

Burn wounds can be caused by exposure to flames, liquids, chemicals, hot surfaces and radiations [48]. Burn introduced direct damage of the mechanical barrier as well as the immune cells in the wound area. The impaired immune response following burns, led to a delay in the healing process and inability to fight infection [29].

A comparison between *Nigella sativa* oil (NSO) and silver sulfadiazine (SSD), the gold standard of burn wound management, was done in the rat model of burn wound healing. NSO-treated group shows faster scab formation with better epithelial appearances in comparison to the SSD group and the control group. NSO-treated group also demonstrated better tissue granulation compared to other groups [34].

In terms of thymoquinone, Selçuk et al. (2013) compared the effect of topical and systemic delivery of thymoquinone in the treatment of burn wound. The results of the study suggested better outcome with topical delivery as compared to systemic delivery of thymoquinone. This is supported by better vascularization and granulation tissue formation, as well as lower inflammatory cell infiltration in the histological assessment of the tissue of the topical treatment groups. However, in combination, topical and systemic treatment of thymoquinone was more superior to all other treatment groups in the study [29].

Positive effect of *Nigella sativa* on wound healing was also apparent in the honey and propolis produced with its nectar. Honey produced by bees has been reported to positively influence EMT of wound healing, and their effect is dependent on the source of nectar [32]. *Nigella sativa* honey, together with ethanolic extract of its propolis demonstrated antibacterial activity and significant promotion of burn wound healing in comparison to SSD cream [48].

The results from these studies supported the superiority of *Nigella sativa* and thymoquinone compared to SSD in the management of burn wound healing.

#### Cutaneous diabetic wound healing

One of the complications of diabetes mellitus is delayed or non-healing wound. This is primarily associated with the series of macrovascular and microvascular alteration that resulted in complications such as poor circulation, weakened immunity, and disrupted cellular metabolism [50].

In diabetic wound, thymoquinone was shown to cause slower overall wound contraction compared to the control. However, upon histological examination at different time point, it was found that thymoquinone induced faster healing at early time point (Day 3) before the healing rate decreases gradually. Observation of lesser inflammatory cell infiltration at Day 3 suggested that thymoquinone enhanced wound healing during the inflammatory phase but started to slow down as the healing shift to proliferative phase. The known anti-angiogenic properties of thymoquinone was believed to be the reason why the healing was slower at the proliferative phase [26].

#### Corneal wound healing

Compared to the cutaneous wound healing, little is known about the association of corneal wound healing with the type 2 EMT. Exposure of corneal epithelial cells to formaldehyde (FA) is known to induce ocular injury [51]. In terms of corneal wound healing, study conducted with FA-exposed corneal keratocyte demonstrated NSO ability to ameliorate inflammatory response produced by FA exposure and restored disrupted corneal architecture. NSO also ensure continuity of limbal stem cells to ensure corneal reepithelialisation [27].

In terms of wound healing, all studies included in the review demonstrated a strong evidence of positive effect of *Nigella sativa* and thymoquinone. Treatment of both *Nigella sativa* and thymoquinone promotes EMT-related pathological changes in the wounded tissue.

### Potential of *Nigella sativa* and its constituent in organ fibrosis

#### Myocardial fibrosis

Myocardial fibrosis contributes substantially to the pathogenesis of heart failure [52]. Prevention of myocardial fibrosis could be beneficial in the management of heart failure. Doxorubicin, a chemotherapy drug that is known to induce severe cardiotoxicity [53] was used to induce heart failure in rats. Treatment of thymoquinone has been shown to have anti-fibrotic and anti-apoptosis effect in this study. The study revealed the potential of thymoquinone as cardioprotective agent for myocardial fibrosis [38].

#### Pulmonary fibrosis

Pulmonary fibrosis is a progressive lung disease that is characterized by destruction of alveolar structures with an abnormal formation of fibroblasts or myofibroblasts and exaggerated synthesis and deposition of extracellular matrix. It can be the result of exposure to environmental contaminant such as paraquat or side effects of chemotherapy drug such as bleomycin.

Pourgholamhossein et al. (2016) conducted the study on preventive and therapeutic value of thymoquinone against paraquat-induced pulmonary fibrosis in rat models. Histologically, thymoquinone treatment restores the inflammatory reaction seen in lung tissue as well as the high hydroxyproline content due to paraquat induction. Thymoquinone treatment also reverses the increase in lipid peroxidation and reduction of antioxidant markers induced by the paraquat. Hydroxyproline content were also significantly reduced and oxidative stress parameter improved. The increase of collagen 1α1, collagen 4α1, and alpha smooth muscle actin (α-SMA) mRNA expression induced by paraquat were also reversed with thymoquinone, justifying the reduction of collagen fibres via Mason Trichrome in the tissue sample [42].

Pulmonary fibrosis can also be the side effects of the drug bleomycin. Another study using bleomycin-induced pulmonary fibrosis in rats demonstrated the beneficial effects of NSO. Bleomycin-induced group shows a disruption in lung architecture, alveolar wall thickening, inflammation and excessive collagen secretion in lung parenchyma. These abnormal changes were restored to normal upon NSO treatment. Tumour growth factor beta 1 (TGF-β1) were also seen to be reduced with NSO treatment [23].

Taken together, outcome from both studies revealed the protective effect of *Nigella sativa* and thymoquinone against lung damage and fibrosis. This suggest the potential of thymoquinone as a treatment modality against pulmonary fibrosis.

#### Hepatic fibrosis

Hepatic fibrosis is the result of physiological response towards chronic liver inflammation that ultimately results in the progressive deposition of ECM and deformation of normal liver architecture.

Effect of monotherapy of thymoquinone or cholecalciferol (vitamin D3) as well as their combination treatment, was investigated by Abdelghany et al. (2016) against carbon tetrachloride (CCL_4_)-induced liver fibrosis in rats. Thymoquinone alone has been shown to resolve liver fibrosis, with better outcome when used together with vitamin D3. The combination therapy resulted in the restoration of liver architecture that is near to normal with less cell infiltration. Serum liver enzymes, namely alanine transaminase (ALT), alkaline phosphatase (ALP) and aspartate transaminase (AST) were also reduced in the combination therapy group. Reduction of fibrotic and inflammatory markers, TGF-β1, interleukin (IL)-6 and IL-22, further supports the anti-fibrotic and anti-inflammatory properties of thymoquinone [40].

#### Renal fibrosis

Renal fibrosis is the inevitable consequence following chronic kidney damage, characterized by the excessive accumulation of extracellular matrix in the kidney parenchyma*.* Fibrosis in the kidney can be detrimental and inevitably leading to renal function deterioration.

NSO has been shown to be renal protective in sodium nitrite-induced kidney toxicity rat model. Nitrite can come from dietary sources as they are used as food preservatives and in processed meats. Physiologically, NSO countered sodium nitrite-induced kidney toxicity by lowering the serum urea and creatinine. Morphologically, NSO demonstrated restoration of the sodium nitrite-induced collagen fibres deposition and disrupted renal parenchyma. In terms of oxidative activity, NSO suppressed oxidative markers and stimulates the production of anti-oxidative markers. NSO also supressed the expression of apoptotic markers. In conclusion, NSO resolved sodium nitrite-induced nephrotoxicity via its anti-oxidative, anti-fibrotic and anti-apoptotic properties [24].

### Potential of *Nigella sativa* and its constituent in inflammation

#### Rhinosinusitis

Rhinosinusitis is a common disorder that is characterized by inflammation involving the mucosa of the nose and paranasal sinuses. Recent study on the association of tissue remodelling in rhinosinusitis verified the involvement of EMT and its associated features such as cell motility in nasal epithelial cells [54]. From the literature search, one study reported the beneficial effect of thymoquinone in resolving experimentally induced-rhinosinusitis in rats [46]. Treatment of thymoquinone improve vascular, inflammatory, and epithelial histological score in inflamed paranasal tissue of rats.

#### Lung inflammation

The lung is exposed to many environmental contaminants, considering its function of filtering inhaled air. Inflammation is the physiological response towards external insult such as the environmental contaminant cadmium. Inflammation in the lung can also be the results of allergic reaction towards common allergen such as ovalbumin.

Protective effect of thymoquinone against cadmium-induced lung inflammation was reported by El-Ebiary et al. (2016). The result remains consistent with previous studies as thymoquinone ameliorated cadmium-induced lung inflammation. Abnormal collagen precipitation and thick intra-alveolar wall were diminished due to anti-inflammatory, anti-fibrotic and anti-oxidative properties of thymoquinone [33].

Su et al. (2016) utilized ovalbumin-induced lung injury in rats to study the beneficial effects of thymoquinone. Ovalbumin induced production of inflammatory markers such as IL-4 and IL-5. Treatment of thymoquinone was reported to reduce the inflammation as evident by the decrease in the inflammatory markers level [43].

In terms of managing lung inflammation, potential of thymoquinone to be used as a treatment is supported by both studies mentioned above. Thymoquinone protects the lung from damage via its anti-inflammatory properties.

#### Nephropathy

Hammad & Lubbad (2016) conducted study on renal ischemic reperfusion injury (IRI) in vivo. Renal arteries were clamped for 35 min and released back to allow reperfusion. This insult results in inflammatory response accompanied by the reduction in renal function parameters. Treatment with thymoquinone increases renal blood flow, and glomerular filtration rate [41].

The effect of thymoquinone in diabetic-induced nephropathy was investigated using streptozotocin-induced diabetic rat model. Glycogen level and urinary albumin excretion were elevated with the induction of streptozotocin. This is followed by the suppression of podocin, a marker for podocyte, the cell that is crucial in glomerular function. Treatment of thymoquinone significantly reduced glycogen and urinary albumin level, as well as restoring the podocin expression. Fibrotic markers, such as TGF-β1 and collagen IV level were also decreased upon thymoquinone treatment. Thymoquinone reduced diabetic nephropathy via the preservation of podocytes and suppression of fibrosis [47].

An earlier study with streptozotocin-induced diabetic rats also shows anti-fibrotic properties of thymoquinone via the increase of epithelial markers, zona occludens (ZO)-1 and reduced mesenchymal markers, FSP1, matrix metalloproteinase (MMP)-17. Histologically, thymoquinone also decreases focal glomerulosclerosis, basal membrane thickening and hyaline deposition in the affected renal tissue sample [45].

#### Liver inflammation

Liver inflammation can be the direct response towards damage-inducing activity to the liver such as chronic alcohol consumption. Using ethanol-induced rat model of liver injury, thymoquinone treatment has been reported to reverse the pathological changes induced by the chronic ethanol consumption. Thymoquinone also demonstrates reduced triglyceride and liver enzyme (ALT and AST) level. Thymoquinone also reduced α-SMA and collagen-I expression. Taken together, thymoquinone protects the liver from inflammation injury via its hepatoprotective properties [44].

## Discussion

The findings from this systematic review highlighted the beneficial effect of black cumin in epithelial to mesenchymal transition (EMT) of type 2. In the context of type 2 EMT, progression in wound healing is dependent upon the attenuation of inflammation while progression of tissue fibrosis is dependent upon the sustenance of inflammation [31]. Hence, reports on enhancement of wound healing and attenuation of both tissue fibrosis and inflammation by black cumin in this review, support the use of this herbal medicine in these different context of type 2 EMT.

The active compound of black cumin, thymoquinone, has been used by 64% of the studies included [25, 26, 29, 36–46]. This enables comparison of its effect between the different studies. In the context of wound healing, thymoquinone successfully shown enhancement of wound healing in cutaneous incisional [25], burn [29], and diabetic wound [26]. All three researchers attributed the anti-inflammatory properties of thymoquinone, as evident by the low inflammatory cell infiltration in the respective rats’ tissue biopsy following treatment of thymoquinone, as major contributor of the beneficial effect. In addition, Gomaa et al. (2017) and Selcuk et al. (2017) both mentions that the effect of thymoquinone is not evident during the proliferation phase of the wound healing due to its anti-angiogenic properties.

In the context of tissue/organ fibrosis, thymoquinone was studied using myocardial, pulmonary, and liver fibrosis rat model. Using a multitude of fibrosis markers specific to each organ that they studied, all three researchers were able to demonstrate reversal of the fibrosis with the treatment of thymoquinone. Fibrosis is the accumulation of excess fibrous connective tissue in an organ or tissue in a reparative or reactive process. Prolonged inflammation is what typically drive a healing tissue into the fibrotic tissue. Anti-inflammatory properties of thymoquinone prevents this event, resulting in a halt of the fibrous connective tissue formation.

All of the inflammation studies included in this review reported the anti-inflammatory effect of thymoquinone. Among the experimental models used include rhinosinusitis [46], otitis [36], lung injury [33, 39, 43], nephropathy [41, 45, 47], liver injury [44], and testicular damage [37]. In agreement with all the findings previously mentioned, thymoquinone exhibit anti-inflammatory properties in all tissues and organs studied. Thymoquinone attenuates inflammation through the down-regulation of EMT-related pathology that resulted in the reduction of the inflammatory mediators observed in all studies included.

Traditionally, black cumin is consumed as an oil supplement. This fact probably motivates 27% of the studies included in this review, to use *Nigella sativa* oil as their investigational product of choice [23, 24, 27, 33, 34]. *Nigella sativa* oil demonstrates superior wound healing enhancement in both cutaneous burn wound and corneal abrasion. Once again, the anti-inflammatory property of *Nigella sativa* was highlighted when significant reduction of inflammatory cells can be observed in tissue section isolated from the experimental rats. In the context of organ/tissue fibrosis, *Nigella sativa* oil reverses the fibrotic process induced by bleomycin in the lung and sodium nitrite in the kidney.

From the literature search, most studies on *Nigella sativa* and thymoquinone found were related to cancer. As a result, majority of the studies on *Nigella sativa* and thymoquinone discussed the type 3 EMT [55–58]. In terms of type 2 EMT, scarce amount of literature is available. Consequently, the underlying molecular mechanism of *Nigella sativa* and thymoquinone on EMT type 2 is still largely remained unknown. Hence, more studies need to be done in the future. In this review, studies reporting direct stimulation of EMT type 2 by *Nigella sativa* and thymoquinone were discussed.

Data collected throughout this review shows that *Nigella sativa* and its bioactive compound, thymoquinone helped in accelerating the wound healing process, attenuating inflammation and preventing organ fibrosis via the regulation of the EMT process.

## Conclusion

The studies that were included in this review reported beneficial effect of *Nigella sativa* and its bioactive compound, thymoquinone on type 2 EMT events, particularly wound healing, organ fibrosis, and inflammation. *Nigella sativa* and thymoquinone enhanced EMT-related pathological change in wound healing. In terms of inflammation and fibrosis, *Nigella sativa* and thymoquinone attenuates the EMT-related pathological change. Further studies are needed to be done to further understand the mechanism of action of these empirical evidences.

## Data Availability

Not applicable.

## Abbreviations

ALT: Alanine transferase
AST: Aspartate transferase
CCL_4_: Carbon tetrachloride
DDR2: Discoidin domain receptor tyrosine kinase 2
ECM: Extracellular matrix
EMT: Epithelial to mesenchymal transition
FA: Formaldehyde
FSP1: Fibroblast specific protein-1
NSC: *Nigella sativa* cream
NSO: *Nigella sativa* oil
SSD: Silver sulfadiazine
TGF-β1: Tumour growth factor beta 1
α-SMA: Alpha smooth muscle actin
